# Thoracic Radiofrequency Ablation in a Patient With Von Willebrand’s Disease: A Case Report

**DOI:** 10.7759/cureus.70102

**Published:** 2024-09-24

**Authors:** William J Brandt, Tatiana J Han, Giustino Varrassi, Sahar Shekoohi, Alan D Kaye

**Affiliations:** 1 School of Medicine, Louisiana State University Health Sciences Center, Shreveport, USA; 2 Internal Medicine, Feist-Weiller Cancer Center, Shreveport, USA; 3 Pain Medicine, Paolo Procacci Foundation, Rome, ITA; 4 Anesthesiology, Louisiana State University Health Sciences Center, Shreveport, USA

**Keywords:** ablation, interventional pain, radiofrequency, thoracic medial branch blocks, von willebrand’s disease

## Abstract

Congenital bleeding disorders involve specific deficiencies in factors that can alter hemostasis, increasing the risk of bleeding. This case report describes a patient with Von Willebrand’s disease who was diagnosed with severe thoracic facet arthritis with pain scores of 9/10. An antihemophilic factor (vWF, Humate-P) injection was administered by a hematologist just before thoracic medial branch blocks. Rather than receiving traditional second thoracic medial branch blocks on another day, the patient, after documenting complete relief of his symptoms with 1% lidocaine, received bilateral T6-8 thoracic radiofrequency ablations, which resulted in complete resolution of his symptoms. A second dose of Humate-P was delivered 24 hours post-procedure. Careful planning with hematologists can enable safe and effective interventional pain procedures in patients with congenital hematologic disorders. This appears to be the first case report in world literature of a patient with Von Willebrand’s disease successfully receiving thoracic radiofrequency ablations.

## Introduction

Von Willebrand’s (vWB) disease is a relatively common bleeding disorder, believed to affect approximately 1% of the general population, with clinically significant disease prevalence estimated at around 125 per million people [1]. vWB disease is classified into three distinct types [2]. Type 1, the most common form, follows an autosomal dominant inheritance pattern with poor penetrance and is caused by a partial quantitative deficiency of von Willebrand factor [1]. Type 2 also exhibits an autosomal dominant pattern but is linked to several qualitative defects in the factor [1]. Type 3 is an autosomal recessive disorder characterized by a complete quantitative defect of von Willebrand factor [1]. The typical clinical presentation of vWB disease often includes mucocutaneous bleeding such as epistaxis, menorrhagia, or easy bruising [2]. Factor supplementation, with a half-life of approximately 6-12 hours, is commonly administered to manage bleeding during procedures [3]. Due to the impaired blood clotting process in vWB disease, excessive bleeding is a significant concern, particularly when performing procedures near the spinal cord.

## Case presentation

We present a case of a 32-year-old male presenting with bilateral T6-8 facet arthropathy secondary to a motor vehicle collision in the summer of 2023. The patient was scheduled for a medial branch block of T6-8 as a diagnostic evaluation to treat the pain. However, it was found during a clinic visit that the patient had Type 1 vWB disease. To safely complete the procedure, options were discussed since the patient did not have a significant bleeding history based on his past medical history, and it was decided that our hematology department should evaluate the patient. The patient agreed to be injected with vWB factor preoperatively to reduce the risk of intraoperative bleeding significantly. Shortly after that, with positive verification of pain relief, he underwent bilateral T6-8 radiofrequency ablation on the same day.

**Figure 1:**
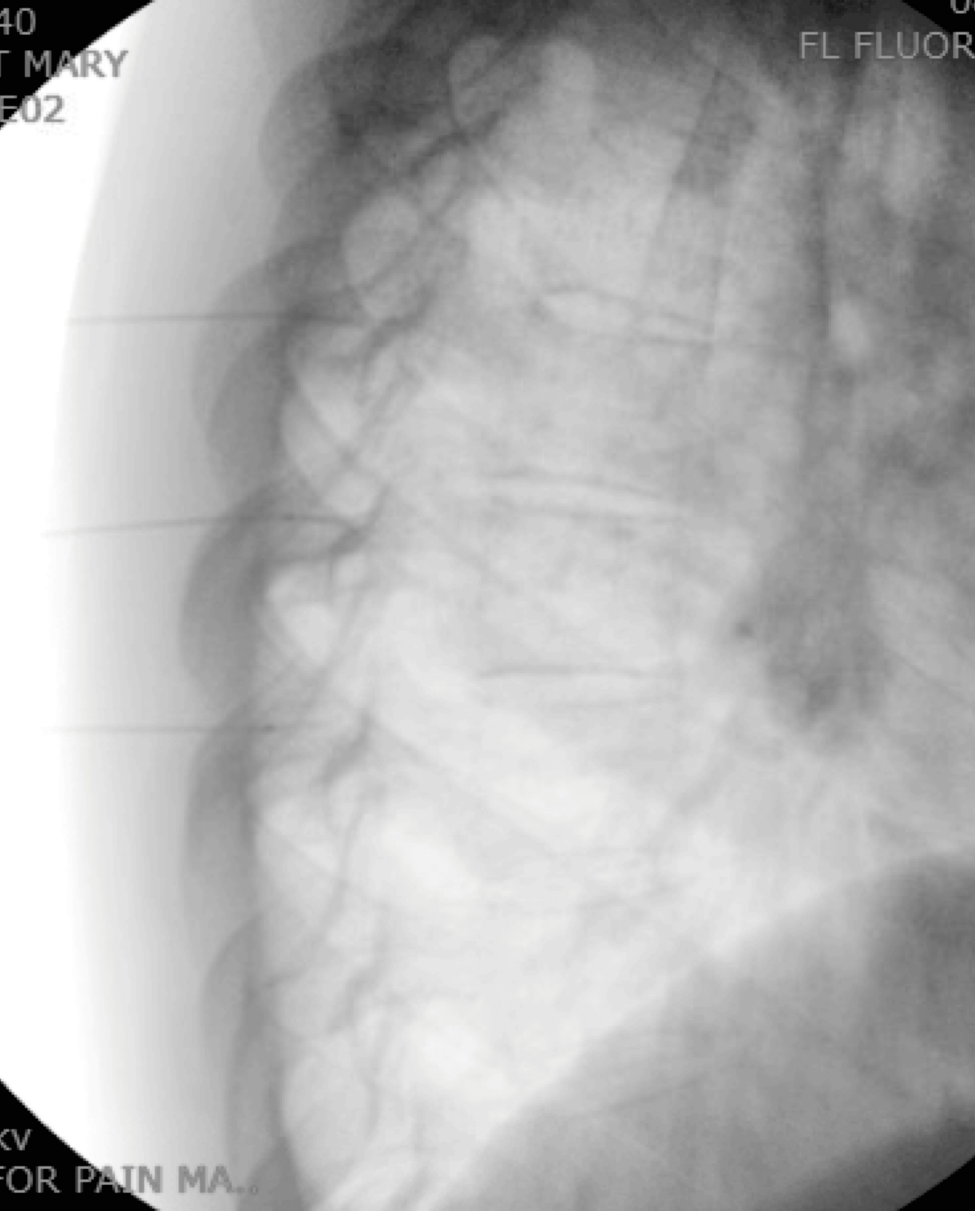
Thoracic medial branch blocks: lateral view.

**Figure 2:**
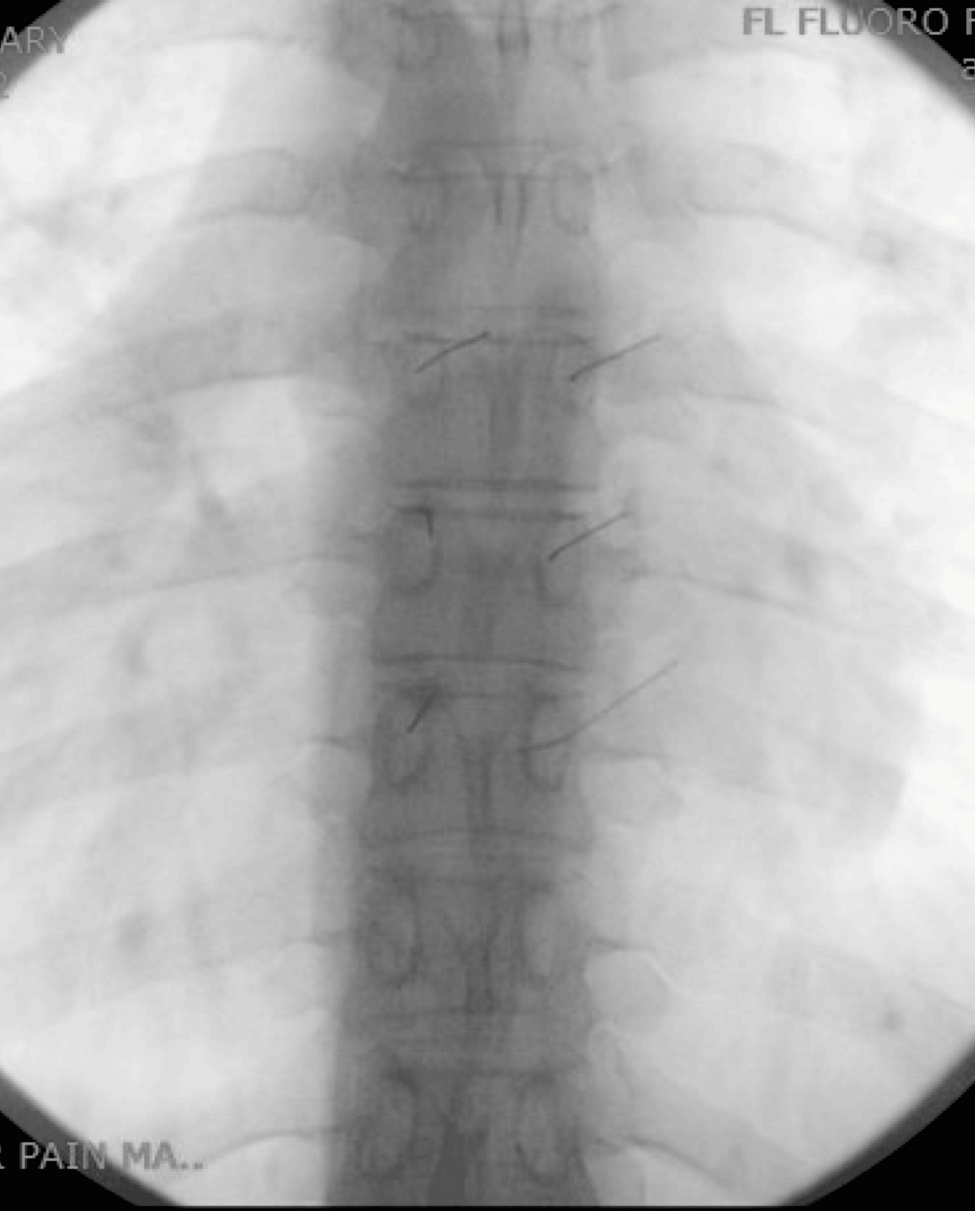
Thoracic medial branch blocks: anterior-posterior view.

After administering the antihemophilic factor-vWF (Humate-P) injection (4000 units over 10 minutes) from our consulting hematologist, we performed a bilateral thoracic medial branch block within the window period. Limited sedation was employed to ensure the patient was fully aware of the improvement in his pain state after the bilateral T6-8 medial branch blocks were performed. After time-out and mild sedation, each site was identified under fluoroscopy. A local anesthetic, 1% lidocaine, was administered, raising a wheal at each level. A 25-gauge, 2.5-inch Quincke needle was advanced to the anatomic location of each medial branch at the junction of the superior articular process and transverse process using intermittent fluoroscopy, as seen in Figure 1. Medication was then injected slowly at a dose of 1 cc of 1% lidocaine. This was repeated on the other side, as seen in Figure 2. The patient was then asked to rate his pain immediately after the procedure. He reported complete resolution of his pain, reducing from 9/10 to 0/10. There was no intraoperative blood loss as the last needle produced less than 0.5 cc of blood. The patient tolerated the procedure well without complications, and diagnostic documentation confirmed thoracic facet arthritis.

**Figure 3:**
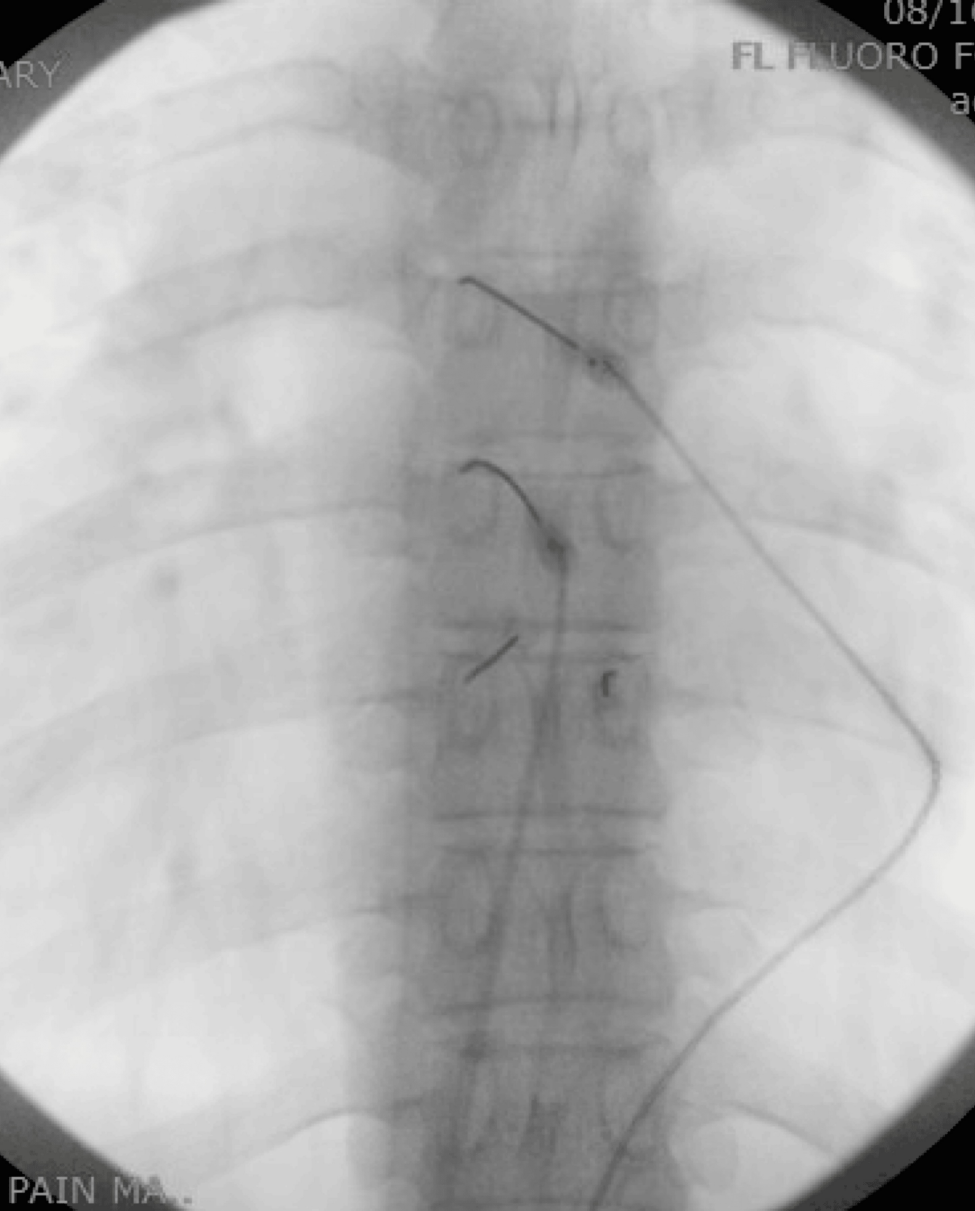
Thoracic radiofrequency ablation: anterior-posterior view.

**Figure 4:**
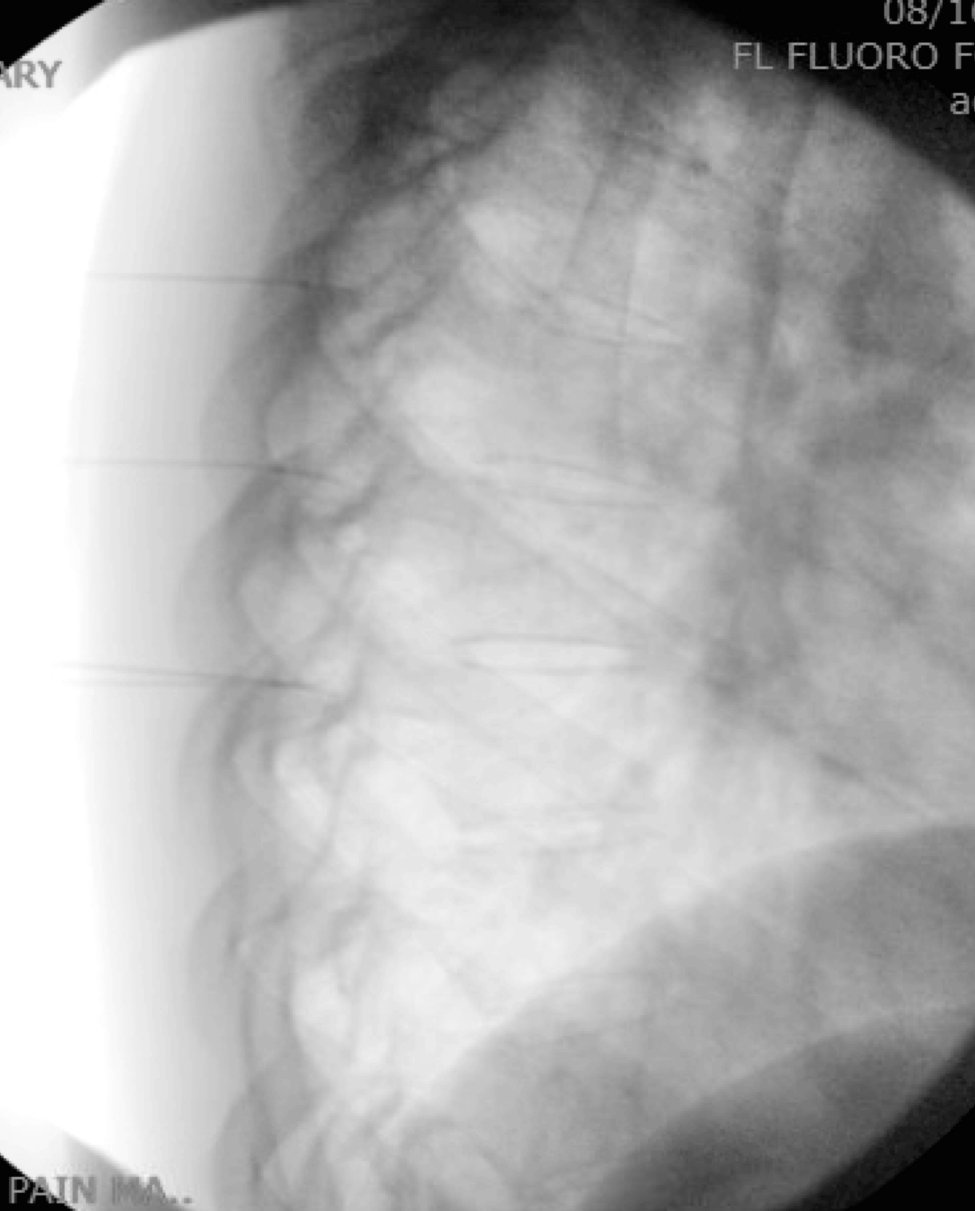
Thoracic radiofrequency ablation: lateral view.

Given that the patient experienced 100% relief with the thoracic medial branch blocks, we proceeded with the radiofrequency ablation (RFA). With the patient still in position and draped, the RFA procedure commenced utilizing an Avanos machine. Each site was identified under fluoroscopy. A local anesthetic with 1% lidocaine was administered by raising a wheal and advancing a 27-gauge, 1.25-inch needle to its hub. A 100 mm, 20-gauge needle was then used for each site and advanced to the anatomic location of each medial branch under intermittent fluoroscopy at each level, as shown in Figures 3, 4.

Motor and sensory testing were performed at each level, with localized sensations limited to the mid-back area. To ensure patient comfort during the radiofrequency ablations, 1 cc of 0.5% ropivacaine was slowly injected at each site. This was repeated on the opposite side. Each ablation was conducted at 80°C for 90 seconds. As with the medial branch block, the patient experienced minimal bleeding during the procedure, with only two needles producing noticeable bleeding of approximately 1 cc in total. The procedure was completed without complications and was well tolerated. Approximately 24 hours post-procedure, the patient received a second dose of antihemophilic factor-vWF (Humate-P) injection, 4000 units, administered over 30 minutes. Consent was obtained through a use-and-disclose-protected health information form from LSU Health Shreveport.

## Discussion

Given the complexities of coordinating with the hematologist and the costs of the synthetic von Willebrand factor, both procedures were consented to initially, allowing us to proceed directly from medial branch blocks to thoracic radiofrequency ablations. In this case, the patient received a total of 8,000 units of antihemophilic factor-vWF (Humate-P), with the cost for each dose being $23,040. The total cost for both doses was approximately $52,000. It is also important to note that the proximity of the lungs increases the risk of pneumothorax and related complications, which could require invasive corrective procedures, significantly raising the chances of bleeding. However, with proper technique and sufficient perioperative infusion of von Willebrand factor, interventional pain procedures on the spine can be safely performed without major blood loss or other complications [4,5]. Complications are always possible in pain management and must be considered as part of the risk assessment before intervention [6,7].

## Conclusions

To the best of our knowledge, this is the first case reported in the literature of a thoracic radiofrequency ablation performed on a patient with von Willebrand’s disease. Patients with von Willebrand’s disease present unique challenges when undergoing medial branch blocks and radiofrequency ablations, particularly in the thoracic cavity. Extra caution must be exercised to avoid entering any vascular beds near the spinal region, which carry a high risk of spinal hematomas. The potential risks associated with desmopressin in epidural steroid injections have been well-documented in the literature. As a result, the decision was made to consult with a hematologist and administer synthetic von Willebrand factor both before the procedure and 24 hours post-procedure.
